# Enhancing brain tumor detection in MRI images through explainable AI using Grad-CAM with Resnet 50

**DOI:** 10.1186/s12880-024-01292-7

**Published:** 2024-05-11

**Authors:** Mohamed Musthafa M, Mahesh T. R, Vinoth Kumar V, Suresh Guluwadi

**Affiliations:** 1 Al-Ameen Engineering College (Autonomous), Erode, Tamil Nadu India; 2 Department of Computer Science and Engineering, JAIN (Deemed-to-be University), Bengaluru, 562112 India; 3 School of Computer Science Engineering and Information Systems, Vellore Institute of Technology University, Vellore, 632014 India; 4 Adama Science and Technology University, Adama, 302120 Ethiopia

**Keywords:** Explainable AI, Brain tumor detection, MRI images, Deep learning, Grad-CAM, ResNet50, Medical image analysis

## Abstract

This study addresses the critical challenge of detecting brain tumors using MRI images, a pivotal task in medical diagnostics that demands high accuracy and interpretability. While deep learning has shown remarkable success in medical image analysis, there remains a substantial need for models that are not only accurate but also interpretable to healthcare professionals. The existing methodologies, predominantly deep learning-based, often act as black boxes, providing little insight into their decision-making process. This research introduces an integrated approach using ResNet50, a deep learning model, combined with Gradient-weighted Class Activation Mapping (Grad-CAM) to offer a transparent and explainable framework for brain tumor detection. We employed a dataset of MRI images, enhanced through data augmentation, to train and validate our model. The results demonstrate a significant improvement in model performance, with a testing accuracy of 98.52% and precision-recall metrics exceeding 98%, showcasing the model’s effectiveness in distinguishing tumor presence. The application of Grad-CAM provides insightful visual explanations, illustrating the model’s focus areas in making predictions. This fusion of high accuracy and explainability holds profound implications for medical diagnostics, offering a pathway towards more reliable and interpretable brain tumor detection tools.

## Introduction

Brain tumors, comprising a range of neoplasms within the brain, pose significant health risks and challenges in medical diagnostics. They are categorized into primary tumors, originating in the brain, and secondary tumors, which metastasize from other body parts. The global incidence of brain tumors underscores a critical need for precise diagnostic tools. Brain tumors exhibit heterogeneous symptoms ranging from headaches to more severe neurological impairments, necessitating early and accurate detection to optimize treatment outcomes [1].

Moreover, the overlapping symptoms of brain tumors with other neurological disorders necessitate a diagnostic tool that offers both high sensitivity and specificity. Traditional diagnostic methods, while effective, often require invasive procedures or can be limited in their ability to detect small or early-stage tumors.

Magnetic Resonance Imaging (MRI) has emerged as a cornerstone in the non-invasive diagnosis of brain tumors [2], offering detailed images of the brain’s anatomy and pathology. MRI provides unparalleled soft tissue contrast, facilitating the distinction between healthy and pathological tissues. It is instrumental in assessing the tumor’s location, size, and potential impact on adjacent brain structures, critical for treatment planning. However, the interpretation of MRI scans is highly reliant on the expertise of radiologists and can be time-consuming, highlighting the need for assistive technologies to improve diagnostic accuracy and efficiency.

**Fig. 1:**
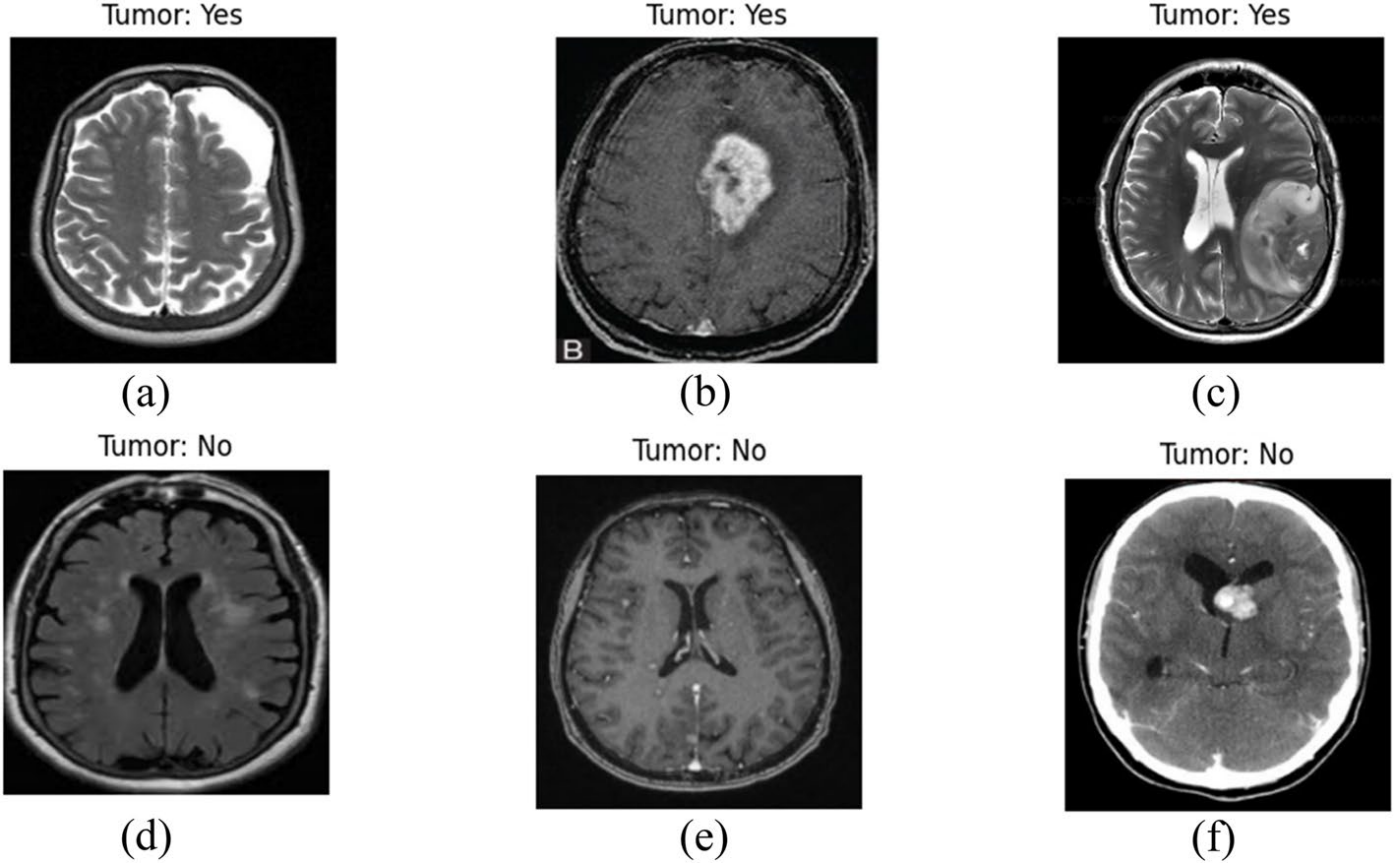
Sample Images from Dataset

Deep learning, a subset of machine learning, has revolutionized the field of medical image analysis, offering substantial improvements in detecting and classifying various diseases [3]. In brain tumor detection, deep learning algorithms can analyze complex MRI data, identify patterns imperceptible to the human eye, and learn from these patterns to make accurate predictions. These algorithms, particularly convolutional neural networks (CNNs), have demonstrated their prowess in enhancing the accuracy and speed of brain tumor diagnostics, reducing the reliance on human interpretation and potentially minimizing diagnostic errors. In Fig. 1, some of visual instances of the brain tumor are shown from the dataset.

The primary objective of this research is to harness the capabilities of deep learning, specifically the ResNet50 architecture, in conjunction with Gradient-weighted Class Activation Mapping (Grad-CAM), to enhance the detection and interpretability of brain tumor diagnoses from MRI scans. This study aims to:

1. Implement a deep learning model that provides state-of-the-art accuracy in detecting brain tumors from MRI images.
2. Integrate Grad-CAM with the deep learning model to offer visual explanations for the model’s predictions, enhancing the interpretability and trustworthiness of the AI system.
3. Evaluate the model’s performance using a comprehensive set of metrics, ensuring its reliability and applicability in a clinical setting.
4. Contribute to the body of knowledge by providing insights into how AI can be made more transparent and assistive in medical diagnostics, particularly in the context of brain tumor detection.

Through these objectives, the study aims to bridge the gap between advanced AI technologies and clinical applicability, offering a tool that not only excels in accuracy but also in providing clarity and insight into its diagnostic processes. This contribution is pivotal in advancing the field of medical diagnostics, where trust and transparency are as crucial as accuracy and efficiency.

## Related work

Brain tumor detection using MRI images has been a focal point of research due to MRI’s capability to provide detailed and high-contrast images. Various traditional image processing techniques, including segmentation and feature extraction, have been employed to differentiate between normal and abnormal brain tissues. However, these methods often require manual intervention and are limited by their reliance on predefined features, which may not capture the full complexity of brain tumor characteristics.

Recent advancements have seen a shift toward automatic brain tumor detection methods, leveraging machine learning algorithms to improve diagnostic accuracy and efficiency. For example, studies have applied Support Vector Machines (SVM) and Random Forest classifiers to MRI data, demonstrating significant success in tumor identification [4]. Yet, these machine learning approaches often require meticulous feature engineering, which can be labor-intensive and may not generalize well across diverse datasets.

Deep learning, particularly convolutional neural networks (CNNs), has revolutionized the field of medical imaging. Unlike traditional machine learning, deep learning eliminates the need for manual feature extraction, allowing the model to learn features directly from the data. This capability has been particularly transformative in brain tumor detection [5], where the intricate and varied nature of tumors necessitates a nuanced analysis.

Studies utilizing deep learning for brain tumor detection have shown remarkable success. CNNs, for instance, have been extensively used to classify and segment brain tumors in MRI scans [6, 7], achieving substantial improvements in accuracy compared to previous methodologies. Some research has also explored the use of transfer learning, where pre-trained models on large datasets are fine-tuned for specific medical imaging tasks, yielding impressive results even with relatively small datasets [8].

Despite these advancements, a critical limitation of deep learning models in medical imaging, particularly in brain tumor detection, is their “black box” nature. The complex architectures of these models make it challenging to understand the reasoning behind their predictions, which is a significant barrier to their acceptance and implementation in clinical settings [9]. In Table 1 a summary of different studies has been given.

**Table 1:** Summary of different studies

Study	Objective	Summary
Patil & Kirange, (2023) [10]	Design a deep ensemble model to improve the accuracy of multiclass classification for brain tumors using MRI scans, addressing the challenges of tumor localization and classification.	Presents a deep ensemble model combining SCNN and VGG16 networks for brain tumor classification from MRI scans, achieving 97.77% accuracy and addressing issues of overfitting and dataset imbalance.
Woźniak et al., (2023) [11]	Introduce a CLM to enhance the efficiency of deep neural network architectures, particularly for evaluating CT brain scans.	Presents a CLM model integrating support neural network with CNN for faster learning and higher efficiency, achieving approximately 96% accuracy in CT brain scan evaluation.
Abdusalomov et al., (2023) [12]	Enhance brain tumor detection using an improved YOLOv7 model with image enhancement, data augmentation, and feature fusion techniques.	Introduces a refined YOLOv7 model for accurate detection of brain tumors in MRI scans, achieving competitive performance and demonstrating potential usefulness in medical applications.
Mahmud et al., (2023) [13]	Develop a CNN architecture for efficient brain tumor detection from MR images, comparing its performance with established models.	Proposes a CNN architecture for brain tumor detection from MR images, achieving superior performance compared to established models with 93.3% accuracy, 98.43% AUC, and 91.19% recall.
Asad et al., (2023) [14]	Develop an automatic system for early detection of brain tumors using a deep CNN with SGD optimization algorithm.	Employs a deep CNN with SGD optimization for brain tumor detection, outperforming baseline methods and suggesting potential for other diseases.
Kanchanamala et al., (2023) [15]	Develop an accurate brain tumor detection and classification system using ExpDHO-based ShCNN and Deep CNN, enhancing accuracy, sensitivity, and specificity.	Proposes an approach combining ExpDHO-based ShCNN and Deep CNN for brain tumor detection and classification, achieving accuracy, sensitivity, and specificity values exceeding 0.9.
Aggarwal et al., (2023) [16]	Develop an improved ResNet-based approach for brain tumor segmentation in MRI images.	Presents an improved ResNet-based approach for brain tumor segmentation, achieving higher precision and accelerating the learning process.
Archana & Komarasamy, (2023) [17]	Evaluate the accuracy of a novel BKNN-based method for brain tumor segmentation in MRI images.	Introduces a BKNN-based method for brain tumor segmentation, aiming to improve accuracy and simplify the segmentation process.
Gayathri et al., (2023) [18]	Assess the effectiveness of the VGG-16 architecture in accurately detecting brain tumors through deep learning.	Evaluates the performance of VGG-16 in brain tumor detection, achieving 94% accuracy after hyperparameter optimization.
Haq et al., (2023) [19]	Develop efficient CNN-based techniques for brain tumor identification and classification from MRI data.	Presents CNN-based techniques for brain tumor identification and classification using MRI data, achieving high accuracy and leveraging conditional random fields for fine segmentation.

While deep learning models have set new benchmarks in the accuracy of brain tumor detection from MRI images, their lack of interpretability remains a significant hurdle. The ability to understand and trust the model’s decision-making process is crucial for clinicians to adopt these AI-assisted diagnostic tools.

Furthermore, many existing studies focus predominantly on model accuracy, often overlooking the aspect of generalizability. It is crucial for models to not only perform well on the data they were trained on but also maintain their performance across diverse and unseen datasets.

This study addresses these gaps by integrating Grad-CAM with a deep learning model, specifically ResNet50, to offer visual explanations for the model’s predictions. Grad-CAM provides a heatmap visualization, highlighting the regions in the MRI images that significantly influence the model’s decision, thereby offering a window into the model’s “thought process.” This approach not only aims to enhance the model’s interpretability but also strives to build trust among clinicians by providing a transparent AI tool that can assist in diagnostic decisions [20].

Additionally, this research emphasizes evaluating the model’s generalizability by testing its performance on a separate, unseen dataset, ensuring that the proposed solution is robust and applicable in real-world clinical settings [21]. Through these efforts, the study aims to contribute a more transparent, understandable, and reliable AI-based tool for brain tumor detection, addressing critical gaps in the current landscape of medical imaging analysis.

## Methodology

**Fig. 2:**
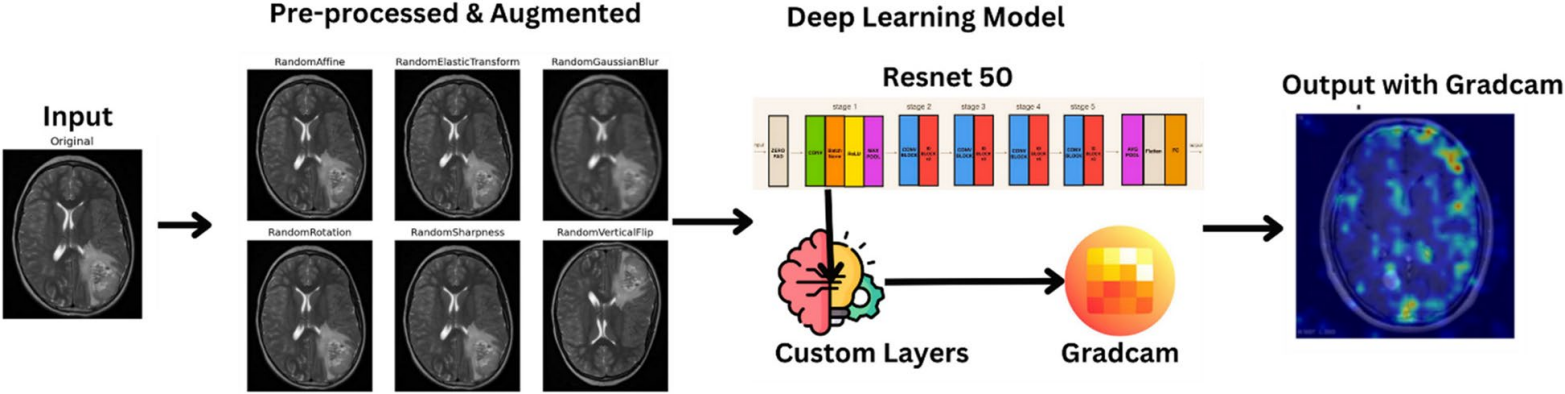
Workflow of the proposed model

The methodology of this study is structured to leverage deep learning for brain tumor detection from MRI images, with a specific focus on enhancing the interpretability of the model using Grad-CAM. This involves a comprehensive process that includes dataset preparation, data preprocessing, model training with ResNet50 [22], application of Grad-CAM for interpretability, and evaluation of the model’s performance. Each step is meticulously designed to ensure that the model not only achieves high accuracy but also provides insights into its decision-making process, crucial for clinical acceptability. Figure 2 depicts the workflow of the proposed model.

### Description of the dataset

**Fig. 3:**
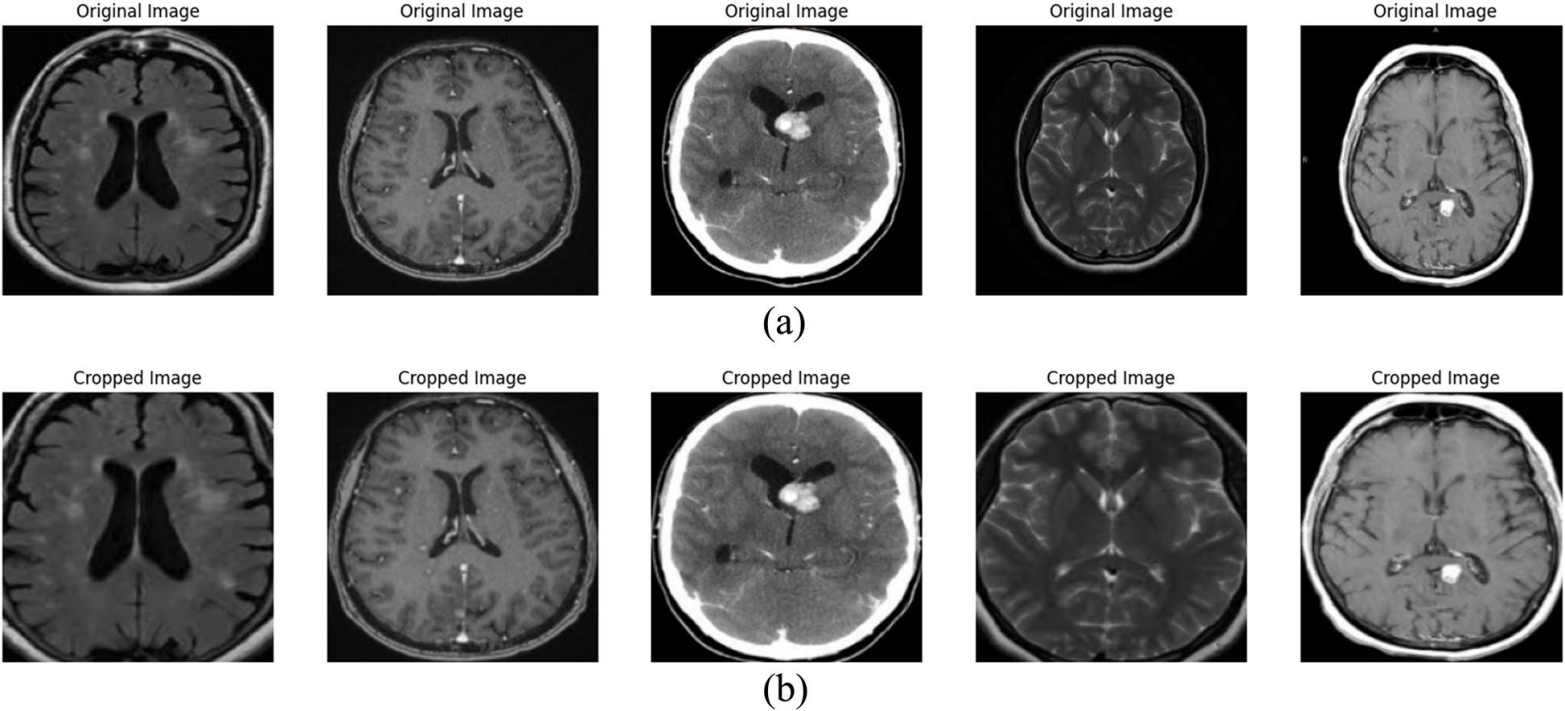
Basic Pre-Processed Image

The dataset used in this study comprises MRI brain images labeled as ‘tumor’ or ‘no tumor’, facilitating a binary classification task. These images are sourced from a publicly accessible medical imaging dataset [23], ensuring the study’s reproducibility. Each image is annotated by expert radiologists, providing a reliable ground truth for model training and evaluation. Figure 3 shows some basic pre-processed images.

Table 2 provides a summary of the dataset.

**Table 2:** Dataset description

	Original	Augmented
Tumor	155	1240
No Tumor	98	784

**Fig. 4:**
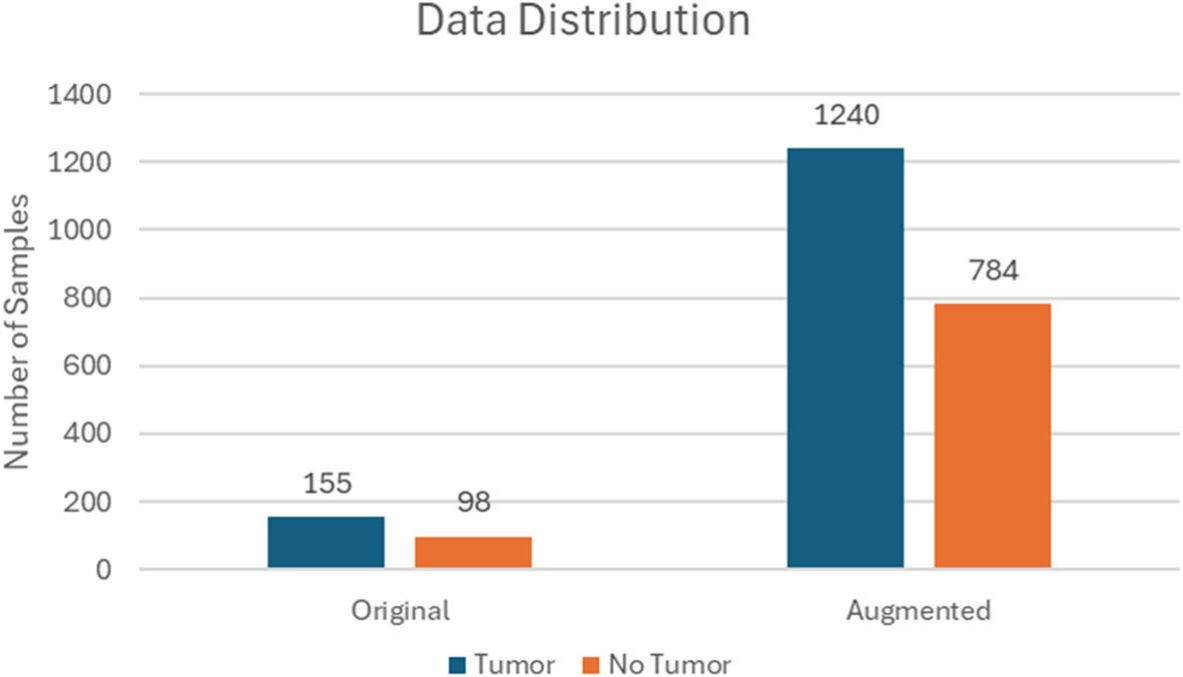
Dataset distribution

Figure 4 presents the data distribution.

The dataset includes a diverse range of images to encompass various tumor types, sizes, and locations, aiming to enhance the model’s generalizability. It contains thousands of images, split into training, validation, and test sets. The training set is used to train the model, the validation set to tune the hyperparameters and prevent overfitting, and the test set to evaluate the model’s performance on unseen data.

### Data preprocessing steps

In the context of neuroimaging research, particularly in the realm of brain tumor analysis, meticulous preprocessing methodologies are fundamental for optimizing the integrity and utility of the dataset utilized for subsequent model training and validation. The initial preprocessing step involves image standardization, whereby all MRI scans are subjected to rigorous resizing and rescaling operations to conform to a standardized dimension and spatial orientation. This ensures homogeneity across the dataset [24], facilitating consistent data processing and feature extraction procedures. Following standardization, intensity normalization (Eq. 1) techniques are applied to recalibrate the intensity values of MRI images onto a uniform scale.

1 $${x}_{\text{norm}}=\frac{x-{\upmu }}{{\upsigma }}$$

By mitigating the influence of inherent variations in imaging parameters, such normalization enhances the model’s sensitivity to subtle anatomical nuances and pathological features, thereby optimizing its discriminatory capacity.

Concomitantly, data cleaning protocols are rigorously executed to eliminate corrupt or extraneous images that may introduce noise or bias into the learning process. This entails comprehensive quality control checks, including the identification and rectification of artifacts such as motion artifacts, scanner-related distortions, or other anomalies that could confound model training. Through meticulous data curation, the integrity and reliability of the dataset are upheld, ensuring that subsequent stages of model development are founded upon a robust and representative data sample.

Furthermore, advanced preprocessing methodologies may encompass the utilization of sophisticated algorithms for image registration, segmentation, and artifact correction. Image registration facilitates the spatial alignment of MRI scans from different subjects or time points onto a common anatomical template, enabling meaningful inter-subject comparisons and longitudinal analyses. Segmentation algorithms delineate regions of interest within the brain, facilitating precise delineation of tumor boundaries and enabling quantitative characterization of tumor morphology and volume. Additionally, artifact correction strategies leverage advanced signal processing techniques to mitigate the effects of noise, distortion, or other imaging artifacts, thereby preserving data fidelity.

The preprocessing of MRI images is a pivotal step in ensuring that the input data is conducive to the learning process of deep learning models. Each preprocessing step is deliberately chosen and applied to optimize the model’s ability to detect brain tumors with high accuracy and reliability.

All MRI images are resized to a standard dimension to ensure uniformity in input size for the model. This is essential because convolutional neural networks (CNNs) require a fixed input size. Rescaling the pixel values to a range of 0 to 1 assists in stabilizing the training process as it normalizes the gradient updates during backpropagation, leading to faster convergence. MRI images can vary in contrast and brightness due to different scanning protocols. Intensity normalization brings all images to a common intensity scale, which helps the model focus on structural information rather than variations caused by the imaging process. This step is crucial for improving the model’s sensitivity to the actual pathological features of brain tumors. The removal of images with artifacts, such as motion blur or scanner-induced noise, is necessary to prevent the model from learning irrelevant or misleading features. Clean datasets enhance the model’s ability to generalize by learning from high-quality, artifact-free images. Data augmentation, including rotation, flipping, scaling, and elastic deformations, artificially expands the dataset and introduces a variety of transformations that the model might encounter in real-world scenarios. This is particularly important for medical imaging tasks, as it simulates variability in tumor appearance and location, thereby enhancing the model’s robustness and ability to generalize.

The expected impact of these preprocessing steps on model performance is multifaceted. Primarily, they aim to improve the model’s accuracy by providing it with quality data that is representative of the various manifestations of brain tumors. Secondly, these steps help in preventing overfitting by ensuring the model does not learn noise or artifacts, which can be common in medical images. Finally, preprocessing enhances the model’s generalizability, enabling it to perform well across datasets with different imaging characteristics.

The judicious application of these preprocessing steps is anticipated to yield a model that is not only highly accurate in detecting brain tumors but also efficient in training and effective across diverse imaging environments. The uniformity and quality of preprocessed data directly contribute to the model’s learning efficacy, ultimately resulting in a tool that is both reliable and clinically valuable.

### Data Augmentation Techniques

**Fig. 5:**
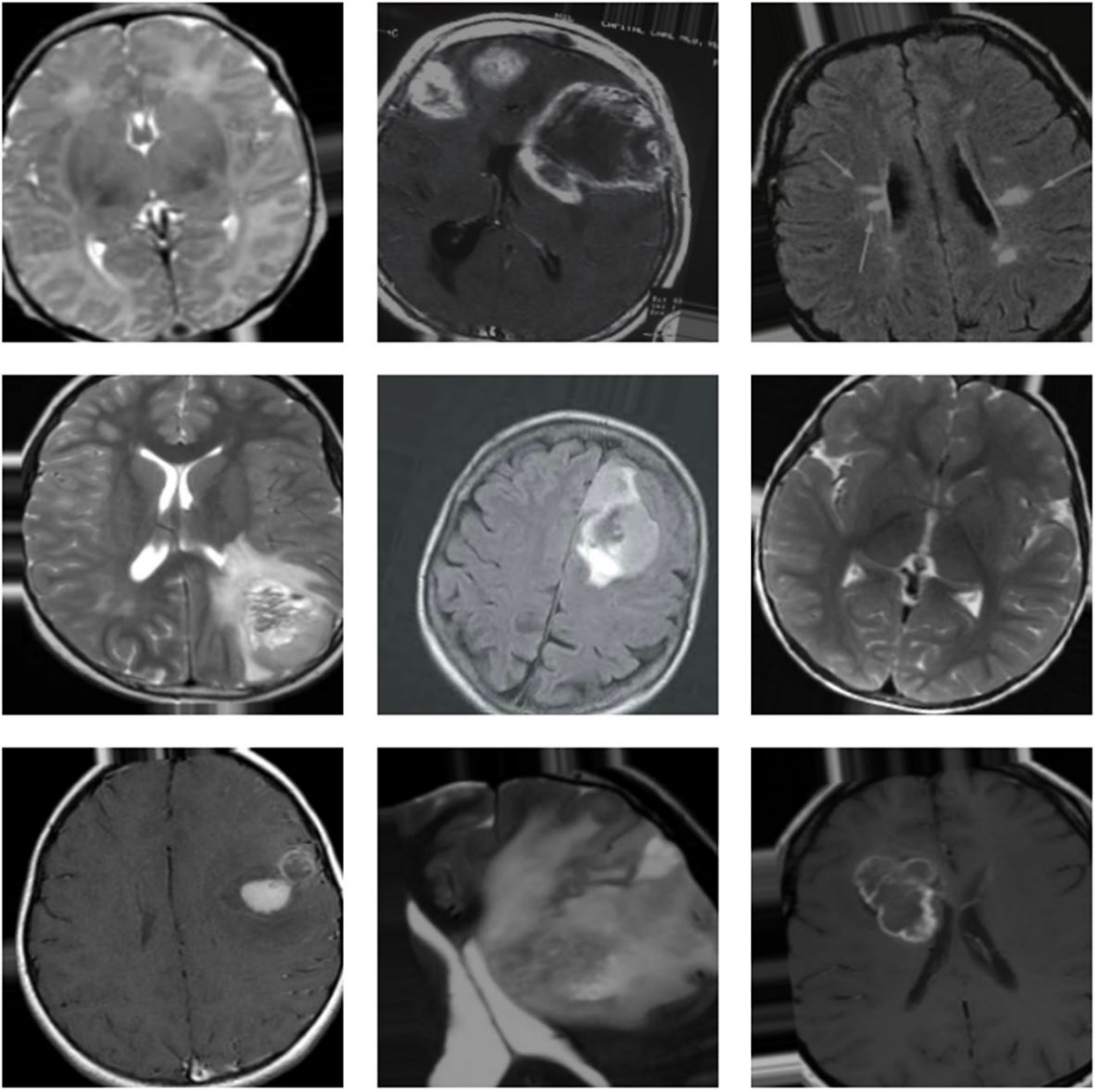
Augmented images

In the domain of brain tumor analysis, where the intricacies and heterogeneity of tumor morphology present significant challenges, the utilization of data augmentation techniques becomes imperative to bolster the dataset’s richness, thereby augmenting the model’s robustness and generalization capabilities. These augmentation methodologies encompass a range of transformative processes, each designed to introduce diverse variations reflective of real-world tumor scenarios. Rotation serves as a foundational technique, facilitating the generation of images from multiple angles to emulate the diverse perspectives encountered in clinical imaging [20]. In Fig. 5 some augmented images are shown.

**Fig. 6:**
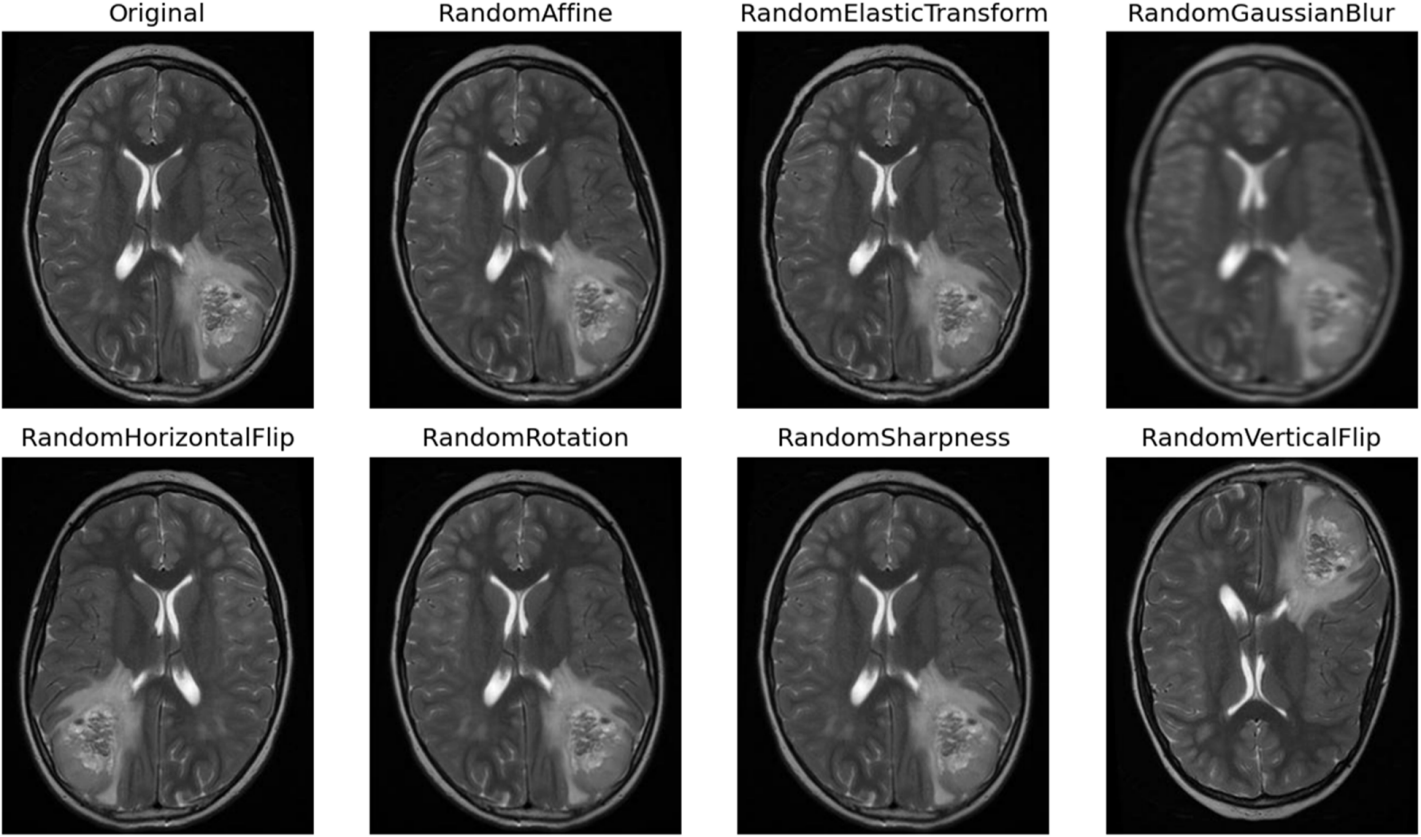
Image after pre-processing steps

In conjunction, flipping operations horizontally and vertically diversify image orientations, effectively mimicking the varying spatial arrangements of tumors within the brain. Scaling manipulations further contribute by resizing images to simulate the spectrum of tumor sizes encountered in clinical practice, thereby enhancing the model’s ability to discern tumors of varying dimensions. Translation operations, both vertically and horizontally, spatially displace images to train the model in tumor localization, regardless of their position within the brain. Figure 6 shows one image after different steps.

Elastic deformation, a sophisticated augmentation technique, introduces realistic distortions to images, thereby emulating the diverse morphological irregularities observed in actual tumor structures. Additionally, adjustments to brightness and contrast levels simulate the range of imaging conditions encountered in clinical settings, ensuring the model’s adaptability to diverse scanning environments. Collectively, these augmentation strategies transcend mere dataset expansion, imbuing the dataset with a comprehensive representation of tumor diversity crucial for robust model training.

In essence, data augmentation serves as a pivotal mechanism for fortifying the model’s resilience and adaptability to the complexities of brain tumor analysis. By encapsulating the myriad manifestations of tumors within the dataset, these augmentation techniques enable the model to generalize effectively across a spectrum of clinical scenarios. Thus, data augmentation emerges not only as a computational strategy but as a fundamental component in refining the diagnostic capabilities of neuroimaging models [25, 26], ultimately advancing the frontiers of brain tumor detection and characterization.

### Overview of the Deep Learning Model (ResNet50) and its relevance

ResNet50 is a variant of the Residual Network (ResNet) architecture, which is designed to train extremely deep neural networks with 50 layers effectively. ResNet introduces the concept of residual learning, which tackles the vanishing gradient problem, allowing the network to learn faster and more effectively, even as the network depth increases. This is achieved through the use of skip connections, or shortcut connections, that allow the gradient to be directly backpropagated to earlier layers.

ResNet50, a prominent convolutional neural network architecture, is characterized by its depth and intricate design elements tailored to facilitate rich feature extraction and gradient propagation. Comprising 50 layers, ResNet50 integrates convolutional layers, rectified linear unit (ReLU) (Eq. 2) activation layers, batch normalization layers, and fully connected layers. Equations 3,10 consists of the various equations used in the model building process.

2 $$f\left(x\right)=\text{max}\left(0,x\right)$$

3 $$\text{Softmax}\left({x}_{i}\right)=\frac{{e}^{{x}_{i}}}{{\sum }_{j}{e}^{{x}_{j}}}$$

4 $$Cross\,Entropy \,Loss=-{\sum }_{i}{y}_{i}\text{log}\left({p}_{i}\right)$$

5 $$Batch \,Normalization=\frac{x-\text{E}\left[x\right]}{\sqrt{\text{Var}\left[x\right]+\upepsilon}}{\upgamma }+{\upbeta }$$

6 $$Residual {x}_{\text{out}}=f\left(x,\left\{{W}_{i}\right\}\right)+x$$

7 $$Adam \,Optimizer \,Update \,Rule \left(First \,Moment\right) {m}_{t}={{\upbeta }}_{1}{m}_{t-1}+\left(1-{{\upbeta }}_{1}\right){g}_{t}$$

8 $$Adam \,Optimizer \,Update \,Rule\left(Second\, Moment\right) {v}_{t}={{\upbeta }}_{2}{v}_{t-1}+\left(1-{{\upbeta }}_{2}\right){g}_{t}^{2}$$

9 $$Adam \,Optimizer \,Weight \,Update {{\uptheta }}_{t+1}={{\uptheta }}_{t}-\frac{{\upeta }{m}_{t}}{\sqrt{{v}_{t}}+\upepsilon}$$

10 $$Learning\, Rate \,Decay {{\upeta }}_{\text{new}}={{\upeta }}_{\text{old}}\times \text{decay}\_\text{rate}$$

This architecture’s fundamental premise revolves around the notion of constructing a deep network capable of acquiring hierarchical representations of input data, essential for intricate pattern recognition tasks. Central to ResNet50’s efficacy are its residual blocks, wherein the input to a block is directly added to its output, thus establishing a shortcut connection. This mechanism alleviates the vanishing gradient predicament by facilitating the unimpeded flow of gradients during backpropagation, thereby enabling the successful training of deep networks.

Furthermore, ResNet50 incorporates a bottleneck design within its blocks to mitigate computational complexity while maintaining efficacy. This design entails employing a sequence of operations within each block: first, a 1 × 1 convolution is utilized to reduce the dimensionality of the input feature maps; subsequently, a 3 × 3 convolution is applied to capture intricate spatial patterns; finally, another 1 × 1 convolution is employed to restore the original dimensionality. By strategically employing these bottleneck structures, ResNet50 optimizes computational efficiency without compromising the network’s capacity to capture complex features, thereby enabling proficient training and inference across diverse applications in computer vision and beyond.

ResNet50 emerges as a pivotal tool owing to its deep architecture and adeptness in extracting intricate features from medical imaging data. Leveraging its hierarchical feature learning capabilities, ResNet50 excels in discerning subtle and complex patterns within MRI images that signify the presence of tumors. Moreover, the application of transfer learning augments its utility in medical imaging tasks, where limited dataset sizes pose challenges for training deep networks from scratch. By pre-training on extensive datasets like ImageNet and subsequently fine-tuning on MRI images, ResNet50 harnesses the knowledge of generic features acquired from larger datasets to adapt to the nuances of tumor detection, thereby enhancing its performance and generalization capacity.

Furthermore, the integration of interpretability techniques such as Gradient-weighted Class Activation Mapping (Grad-CAM) with ResNet50 contributes to its utility in clinical settings. This methodology enables the visualization of salient regions within input images that influence the model’s decision-making process, thereby enhancing interpretability. Clinicians gain insights into the rationale behind the model’s predictions, as Grad-CAM elucidates the areas deemed indicative of tumor presence. This not only bolsters confidence in the model’s diagnoses but also facilitates collaborative decision-making processes between clinicians and AI systems, ultimately enhancing patient care and treatment planning.

ResNet50 is adapted for the binary classification task of detecting brain tumors. The final fully connected layer of the standard ResNet50 model, typically used for 1000-class classification, is replaced with a new layer tailored to distinguish between two classes: ‘tumor’ and ‘no tumor’. This adaptation is crucial for tailoring the pre-trained model to the specific task at hand.

The model is trained on the augmented MRI dataset, leveraging backpropagation to minimize the loss function and update the weights. During training, the effectiveness of ResNet50’s residual blocks is leveraged to capture the intricate details necessary for accurate tumor detection. Grad-CAM is then applied to the trained model, providing visual explanations that highlight the regions in the MRI images most influential to the model’s predictions, thus offering a transparent view into the model’s operational mechanics.

The algorithm 1 provides a structured approach to leveraging ResNet50 combined with Grad-CAM for the task of brain tumor detection from MRI images, emphasizing both accuracy in classification and transparency in model decision-making through visual explanations.

**Figure:**
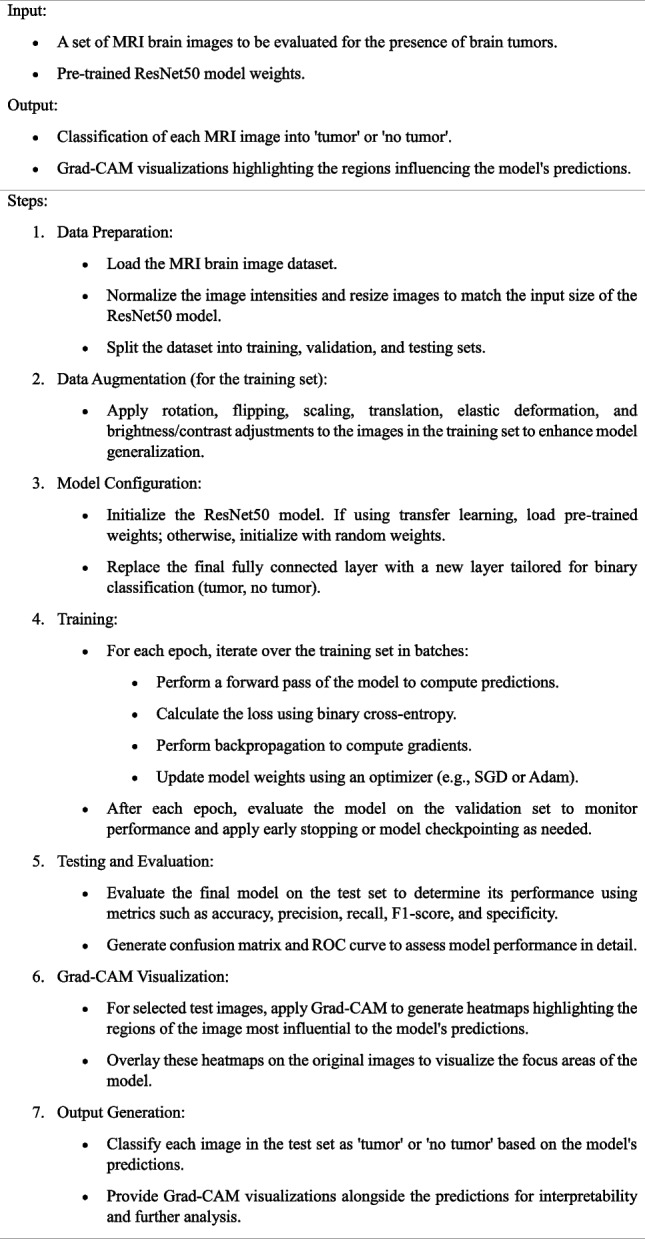
**Algorithm 1.** Brain Tumor Detection Using ResNet50 with Grad-CAM

### Training, validation and testing process

The training process for the ResNet50 model in brain tumor detection entails a meticulous sequence of steps aimed at optimizing its performance and robustness. Commencing with model initialization, pre-trained weights from datasets like ImageNet are often leveraged to kickstart the learning process, facilitating transfer learning and expediting convergence towards task-specific objectives. Subsequently, a suitable loss function, typically binary cross-entropy for binary classification tasks, is selected to quantify the disparity between predicted outputs and actual labels. An optimizer, such as SGD or Adam, is then chosen to iteratively update the network’s weights based on feedback from the loss function.

Batch processing is employed to partition the training dataset into manageable subsets, enabling incremental weight updates and enhancing computational efficiency. During each training iteration, a forward pass propagates data through the network, generating predictions. Following this, a backward pass, known as backpropagation, computes the gradient of the loss with respect to the network weights, facilitating weight updates by the optimizer to minimize the loss.

The training process unfolds over multiple epochs, with each epoch representing a complete pass through the entire training dataset. Techniques like dropout or L2 regularization may be employed to mitigate overfitting, ensuring the model generalizes well to unseen data.

Concurrently, the validation process occurs at the end of each epoch, where the model’s performance is evaluated on a separate validation set. This assessment offers insights into the model’s generalization capabilities and informs hyperparameter tuning decisions. Hyperparameters such as learning rate and batch size are adjusted based on performance metrics such as accuracy, precision, and recall. Furthermore, early stopping criteria may be implemented to halt training if the model’s performance on the validation set deteriorates, mitigating overfitting by preventing the model from learning noise from the training dataset. Through this iterative training and validation regimen, the ResNet50 model is honed to achieve optimal performance and reliability in the task of brain tumor detection.

The testing process for the developed deep learning model in brain tumor detection involves several rigorous steps to ascertain its reliability, effectiveness, and interpretability. Following the completion of training and validation phases, the model undergoes final evaluation using a designated test set comprising data that remains unseen during prior phases. This critical step serves to assess the model’s real-world applicability and performance under novel conditions.

A comprehensive array of performance metrics, including accuracy, precision, recall, F1-score, and confusion matrices, are meticulously computed to quantitatively evaluate the model’s efficacy in detecting brain tumors. Particularly within the realm of medical diagnostics, metrics such as sensitivity and specificity hold significant importance, providing insights into the model’s ability to correctly identify both positive and negative cases.

In addition to quantitative assessments, the interpretability aspect is addressed through the generation of Grad-CAM visualizations for test images. These visualizations offer qualitative insights into the areas of focus within the input images that significantly influence the model’s predictions. Such interpretability is paramount for garnering clinical acceptance, as it furnishes practitioners with comprehensible and trustworthy AI-driven insights.

Moreover, the performance on the test set serves as a robust measure of the model’s generalizability, gauging its potential effectiveness and reliability in real-world clinical settings. Through this meticulous training, validation, and testing process, the deep learning model undergoes refinement to ensure not only accuracy but also reliability and interpretability, thereby aligning with the critical requirements of medical imaging analysis. This comprehensive approach underscores the commitment to delivering robust and clinically applicable solutions in the domain of brain tumor detection.

### Performance metrics used for evaluation

The evaluation of a deep learning model’s performance, such as ResNet50, demands a meticulous selection of performance metrics to ascertain its effectiveness, reliability, and clinical applicability. The chosen metrics play a pivotal role in offering insights into the model’s predictive prowess and its capacity to discern between tumor and non-tumor instances. Accuracy (Eq. 11), defined as the ratio of correctly predicted observations to total observations, provides a fundamental measure of the model’s overall correctness. Precision (Eq. 12), recalling the ratio of correctly predicted positive observations to the total predicted positives, is crucial in medical diagnostics to minimize false positives, while recall (Eq. 13), quantifying the ratio of correctly predicted positive observations to all actual positives, ensures the model’s capability to detect as many true tumor cases as possible. TP is for True Positive, TN is for True Negative, FP is for False Positive and FN is for False Negative.

11 $${Accuracy}=\frac{\text{TP}+\text{TN}}{\text{TP}+\text{FP}+\text{FN}+\text{TN}}$$

12 $$\text{Precision}=\frac{\text{TP}}{\text{TP}+\text{FP}}$$

13 $$\text{Recall}=\frac{\text{TP}}{\text{TP}+\text{FN}}$$

The F1 score (Eq. 14), being the harmonic mean of precision and recall, balances their trade-off and is particularly useful in uneven class distributions. Specificity, delineating the proportion of actual negatives correctly identified, complements recall in ensuring accurate diagnoses by minimizing false negatives.

14 $$F1=2\times \frac{\text{Precision}\times \text{Recall}}{\text{Precision}+\text{Recall}}$$

The confusion matrix, incorporating true positives, false positives, true negatives, and false negatives, provides a holistic view of the model’s performance across different classes. Receiver Operating Characteristic (ROC) curve (Eq. 15) and Area Under the Curve (AUC) offer insights into the model’s ability to distinguish between classes across various threshold settings, crucial in assessing its discriminative capacity. Mean Squared Error (MSE) (Eq. 16) ,Root Mean Squared Error (RMSE) (Eq. 17) & Mean Absolute Error (MAE) (Eq. 18) typically used in regression tasks, provide quantitative insights into the magnitude of the model’s error.

15 $$\text{ROC-AUC}={\int }_{0}^{1}\text{TPR}\left(f\left(T\right)\right)\text{d}\left[\text{FPR}\left(f\left(T\right)\right)\right]$$

16 $$\text{MSE}=\frac{1}{n}\sum\limits_{i=1}^{n}{\left({Y}_{i}-\widehat{{Y}_{i}}\right)}^{2}$$

17 $$\text{RMSE}=\sqrt{\text{MSE}}$$

18 $$\text{MAE}=\frac{1}{n}\sum\limits_{i=1}^{n}\left|{Y}_{i}-\widehat{{Y}_{i}}\right|$$

F2-Score (Eq. 19) and Cohen’s Kappa (Eq. 20) is also calculated further.

19 $$F2=\left(1+{{\upbeta }}^{2}\right)\times \frac{\text{Precision}\times \text{Recall}}{{{\upbeta }}^{2}\times \text{Precision}+\text{Recall}}$$

20 $${\upkappa }=\frac{{P}_{o}-{P}_{e}}{1-{P}_{e}}$$

Additionally, interpretability metrics like Grad-CAM visualizations serve as qualitative assessments, elucidating the model’s focus areas during prediction, thereby validating its decision-making process in a clinical context. Collectively, these highly technical metrics ensure a comprehensive evaluation of the deep learning model’s performance, reinforcing its reliability and efficacy in clinical applications of brain tumor detection from MRI images.

## Experimentation and results

**Fig. 7:**
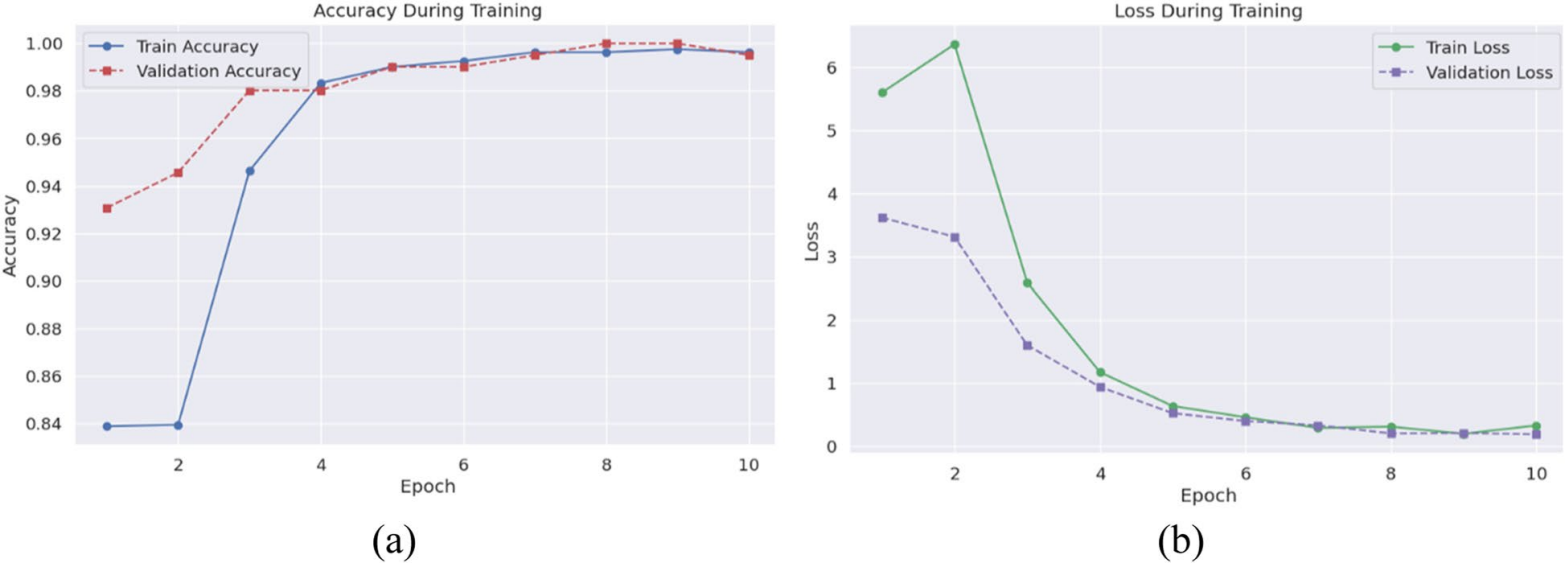
Accuracy and Loss During Training

The deep learning model developed for brain tumor detection underwent rigorous training and testing within a robust computational environment, leveraging Python as the programming language for its extensive machine learning and data processing libraries. PyTorch served as the primary deep learning framework, chosen for its dynamic computation graph and efficient memory utilization, particularly conducive to training complex neural networks like ResNet50. Essential libraries included torchvision for model access and image transformations, PIL for image file operations, NumPy for numerical computations, and matplotlib/Seaborn for visualization. Augmentation techniques significantly expanded the dataset from an initial 253 images to 2024, encompassing rotations, flips, scaling, translations, elastic deformations, and brightness/contrast adjustments, thereby enhancing model generalization and mitigating overfitting risks. The training process spanned 10 epochs, with a batch size of 16 and dynamic learning rate adjustments based on validation set performance. Notable epoch-wise progress showcased consistent improvement, culminating in a test accuracy of 98.52%. In Fig. 7 the training loss and accuracy as per epochs is shown.

Precision and recall metrics for tumor and no-tumor classes exceeded 97% and 98%, respectively, while F1-score averaged around 98%. Interpretability was enhanced through Grad-CAM visualizations, correlating model predictions with radiological markers of brain tumors, reinforcing clinical trust. These comprehensive results underscore the model’s effectiveness in detecting brain tumors from MRI images, elucidating its training process, performance metrics, and interpretive capabilities for clinical adoption. During the validation and testing phases, crucial assessments were conducted to evaluate the model’s performance and its ability to generalize to unseen data. Throughout the validation process, the model demonstrated exceptional accuracy, peaking at 100% by the eighth epoch, affirming its proficiency in classifying MRI images into ‘tumor’ and ‘no tumor’ categories accurately. Precision, recall, and F1-score metrics further underscored the model’s reliability, with values nearing 100% by the eighth epoch, indicative of robust performance in identifying true positive cases while minimizing false positives and negatives. On the test set, the model exhibited an impressive accuracy of 98.52%, complemented by high precision and recall values for both ‘no tumor’ and ‘tumor’ classes, confirming its effectiveness in discerning between pathological and healthy states. Visualization of Grad-CAM results provided an additional interpretive layer to the model’s predictions, generating heatmaps overlaid on MRI images to highlight regions significantly influencing predictions. These visualizations, instrumental for clinicians, validated the model’s attention to clinically relevant features, enhancing trust and reliability. In conclusion, the combination of quantitative metrics and qualitative Grad-CAM visualizations offers a comprehensive evaluation framework, affirming the model’s efficacy in brain tumor detection while ensuring transparency and trustworthiness crucial for clinical adoption.

During the validation and testing phases, the model’s performance and generalization ability were meticulously scrutinized, yielding comprehensive insights into its efficacy in brain tumor detection. Validation accuracy emerged as a pivotal metric, with the model achieving a remarkable peak accuracy of 100% by the eighth epoch, underscoring its proficiency in correctly classifying MRI images into ‘tumor’ and ‘no tumor’ categories. Precision, recall, and F1-score metrics further elucidated the model’s reliability, with precision and recall values nearing 100% by the eighth epoch, translating into an F1-score of approximately 100%. These metrics underscored the model’s capacity to identify true positive cases while minimizing false positives and negatives, essential for precise medical diagnostics. In the subsequent testing phase, the model exhibited a commendable accuracy of 98.52%, slightly lower than the validation accuracy but still indicative of stellar performance. Precision metrics for both ‘no tumor’ and ‘tumor’ classes exceeded 98%, while recall values surpassed 97% and 99%, respectively, affirming the model’s proficiency in correctly identifying actual positive and negative cases. The balanced F1-score around 98% for both classes corroborated the model’s ability to maintain equilibrium between precision and recall, crucial for diagnostic tasks where erroneous classifications carry significant ramifications. Table 3 provides the performance metrics.

**Table 3:** Performance Metrics

	Precision	Recall	F1 Score
No Tumor	0.99	0.97	0.98
Tumor	0.98	0.99	0.99

**Fig. 8:**
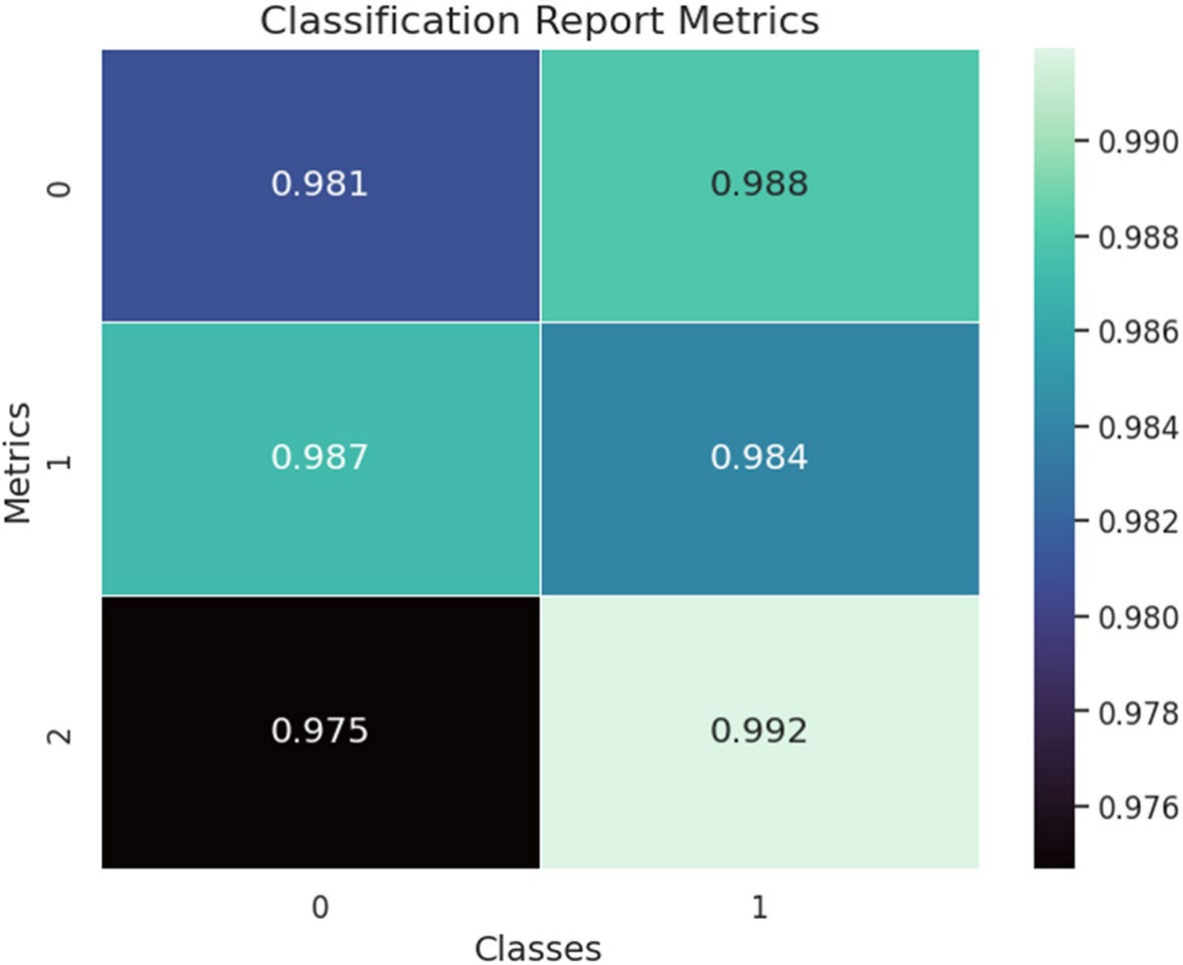
Classification Report

Figure 8 class wise performance metrics is shown.

**Fig. 9:**
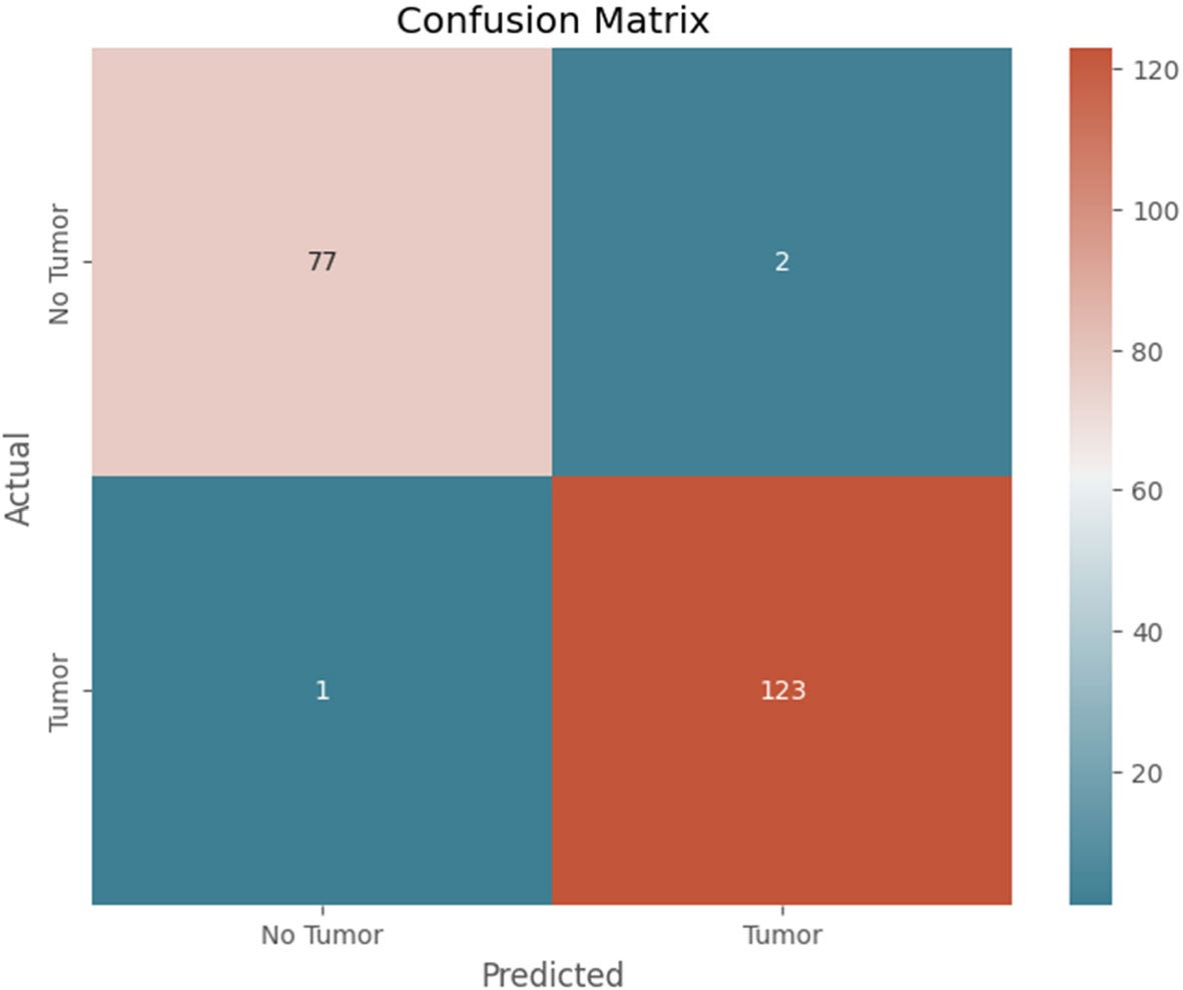
Confusion Matrix

Figure 9 depicts the confusion matrix of the proposed model.

The Table 4 gives insights of the error metrics of the model.

**Table 4:** Error Metrics

Metrics	Value
MSE	0.015
RMSE	0.122
MAE	0.015

**Fig. 10:**
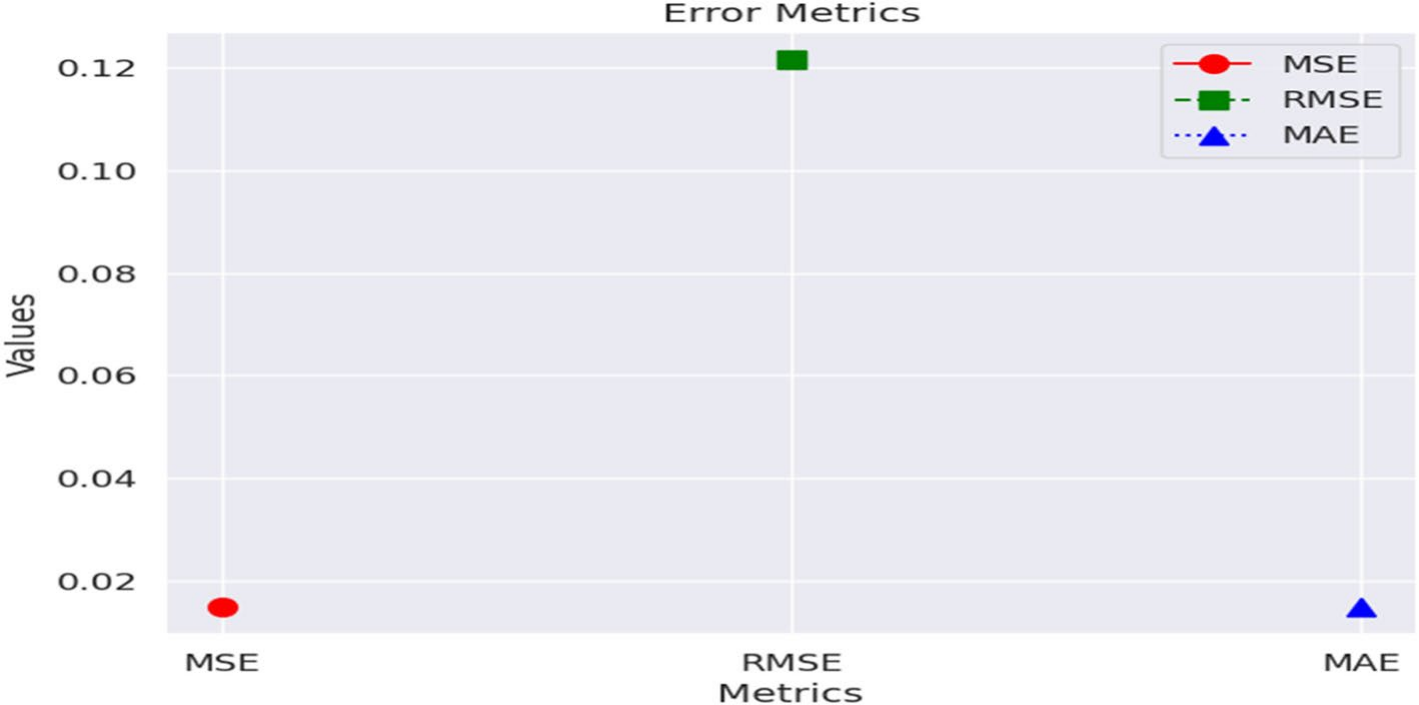
Error Metrics

Figure 10 depicts the Error Metrics of the model.

Cohen’s Kappa and F2 Score value is shown in Table 5.

**Table 5:** Advanced Metrics

Metrics	Value
F2 Score	0.99
Cohen’s Kappa	0.97

**Fig. 11:**
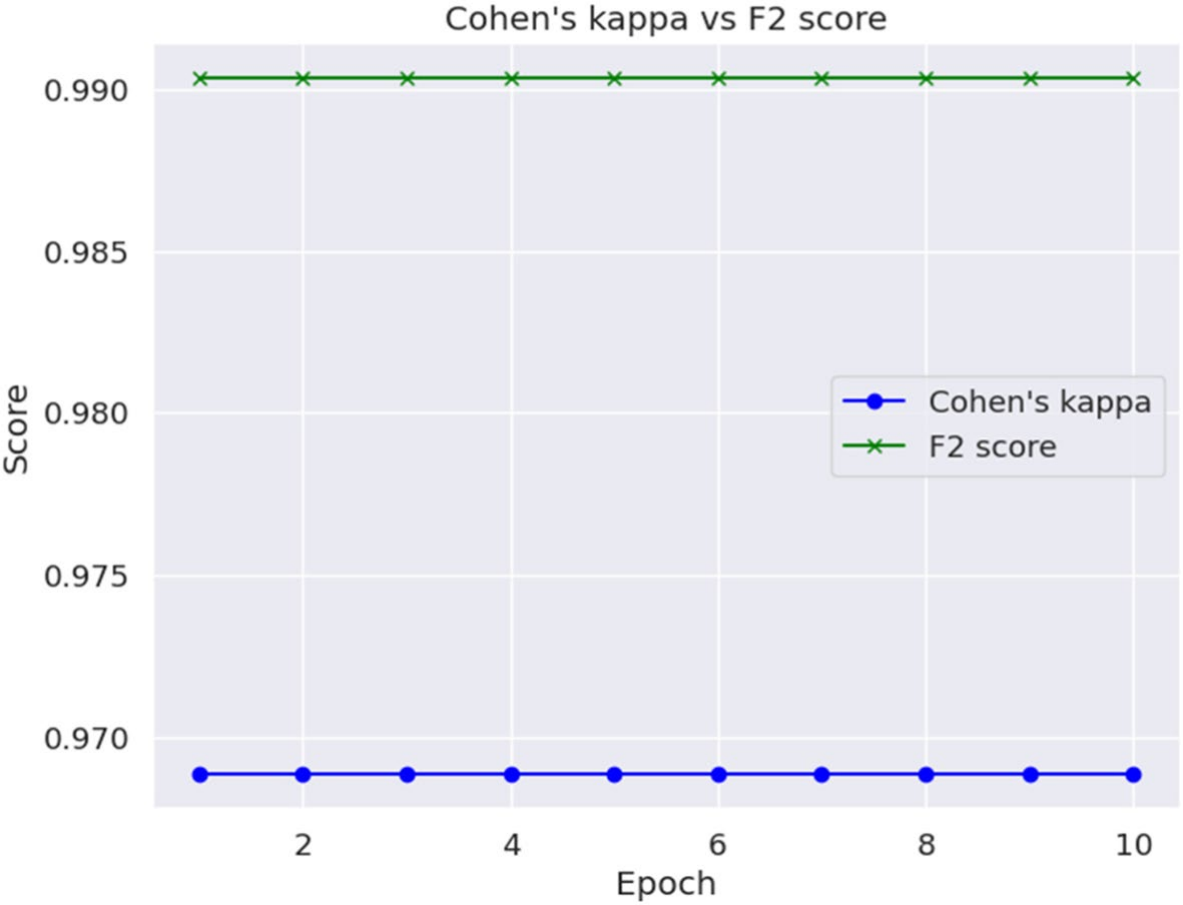
Epoch Wise Cohen’s Kappa and F2 Score

Cohen’s Kappa and F2 Score has been depicted epoch wise in Fig. 11.

**Fig. 12:**
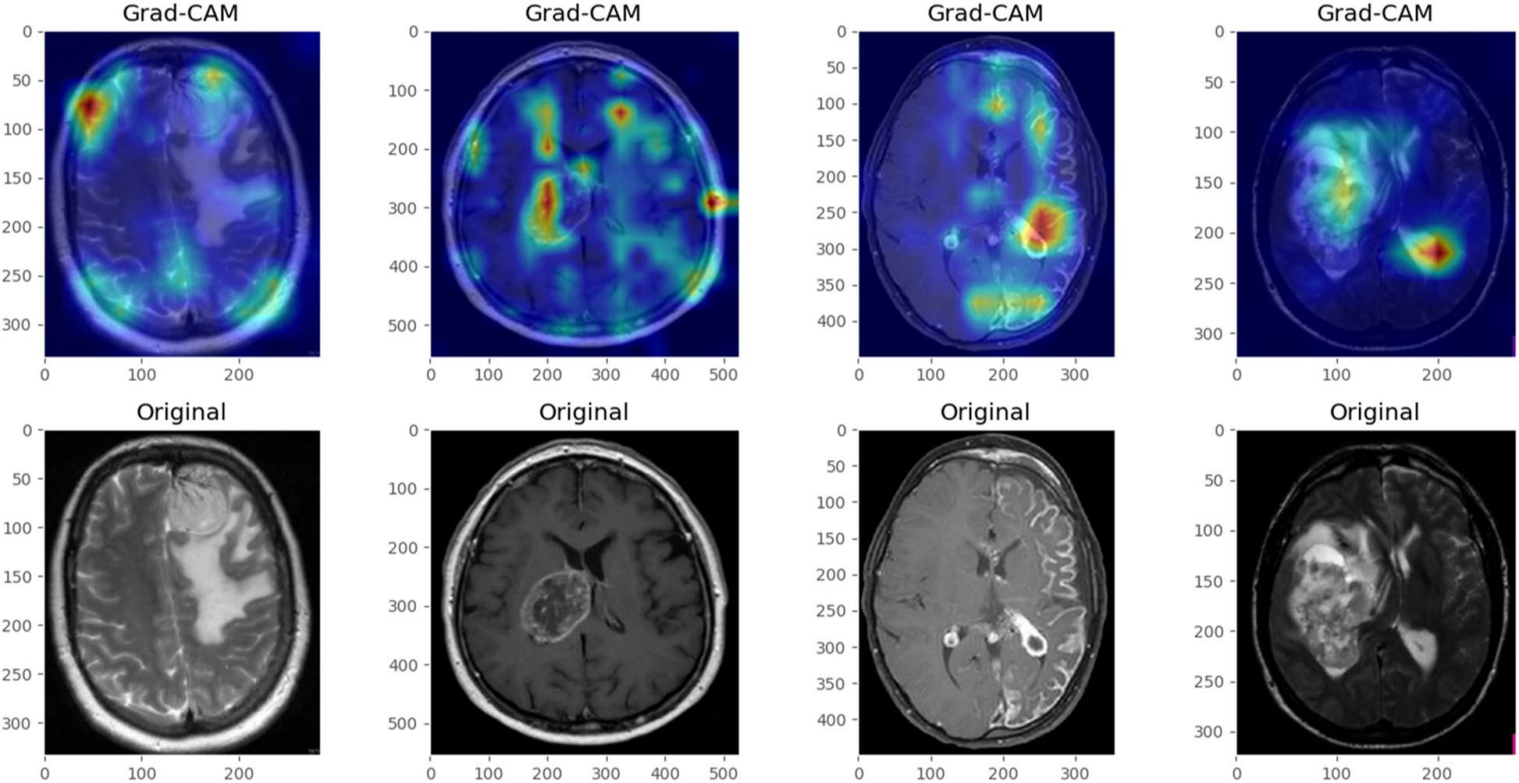
Grad-CAM Visualization

Augmenting the quantitative metrics, the utilization of Gradient-weighted Class Activation Mapping (Grad-CAM) added an interpretive layer to the model’s predictions. Grad-CAM generated heatmaps superimposed on MRI images, spotlighting regions significantly influencing the model’s decisions. In images depicting tumors, the heatmaps predominantly highlighted tumor regions, validating the model’s attention to clinically relevant features. Beyond mere visualization, these heatmaps offered invaluable insights to clinicians, providing a visual affirmation that the model’s decisions were grounded in relevant pathological markers rather than extraneous image features. Such interpretive aids fostered trust and reliability among medical practitioners, ensuring that the model’s decision-making aligned with clinical expectations and knowledge. Figure 12 depicts grad cam visualization of some sample images.

The amalgamation of quantitative metrics and qualitative Grad-CAM visualizations furnished a comprehensive evaluation framework, elucidating the model’s efficacy in brain tumor detection while offering insights into its decision-making rationale. This multifaceted evaluation not only substantiated the model’s diagnostic accuracy but also augmented its transparency and trustworthiness, paramount for garnering acceptance and adoption in clinical settings.

## Discussion

The evaluation of the ResNet50 model, augmented with Grad-CAM for interpretability, in detecting brain tumors from MRI images unveils its outstanding performance and clinical significance. With a testing accuracy reaching 98.52%, the model showcases remarkable robustness and reliability. Precision and recall metrics, soaring to exceptionally high levels, underscore the model’s proficiency in accurately identifying tumor presence while minimizing false diagnoses—a critical aspect in medical contexts where every misdiagnosis carries significant consequences.

When compared with baseline models or previous studies, which often exhibit lower accuracy levels for similar tasks, the ResNet50 model’s performance stands out prominently. Traditional machine learning approaches or earlier deep learning models typically struggle to achieve such high precision and recall levels, particularly in the nuanced and complex task of brain tumor detection from MRI images. The incorporation of Grad-CAM further distinguishes this study, offering a layer of interpretability often absent in conventional approaches. Table 6 comprises of comparison with baseline studies.

**Table 6:** comparison with baseline studies

Study	Technique	Accuracy
Khan et al. (2023) [27]	Brain tumor detection using deep learning	95.94%
Kumar et al. (2023) [28]	Brain tumor classification using CNN models	96.2%
Hossain et al. (2023) [5]	Multiclass brain tumor classification using DL architectures	96.94%
Anaya-Isaza et al. (2023) [29]	Brain tumor classification and detection using DL architectures and Cross-Transformer	97%
Pillai et al. (2023) [30]	Brain tumor detection using deep transfer learning models	91.58%
Sharma et al. (2023) [31]	Brain tumor detection using Modified ResNet50 with HOG features	88%
Pedada et al. (2023) [32]	Brain tumor segmentation using modified U-Net with residual networks	93.40%
Rahman and Islam (2023) [33]	Brain tumor classification using parallel deep convolutional neural network (PDCNN)	97.33%
Proposed Model	Optimized Resnet50 with Gradcam	98.52%

The effectiveness of data augmentation emerges as a pivotal factor in enhancing the model’s generalization capability. By introducing diverse transformations, the model learns to recognize tumors across various presentations, mitigating the risk of overfitting to the training data’s specific characteristics. This holds significant importance in medical imaging, where variability across patients and imaging conditions is ubiquitous.

The selection of ResNet50 as the model architecture significantly contributes to the high performance observed. Its deep layered structure, coupled with residual connections, empowers the model to learn intricate features from MRI images, essential for accurate tumor detection. The success of this architecture in this context reaffirms its efficacy and adaptability to various image recognition tasks, including those in the medical domain.

A critical factor in the adoption of AI-driven diagnostic tools is their ability to provide interpretative outputs that resonate with expert clinical judgment. To this end, the proposed study employs Gradient-weighted Class Activation Mapping (Grad-CAM) to generate visual explanations for the model’s predictions. The previous research works carried out in this field along with the survey’s carried out signifies that grad cam is better for the model’s interpretative visualizations align with expert radiological assessments.

**Fig. 13:**
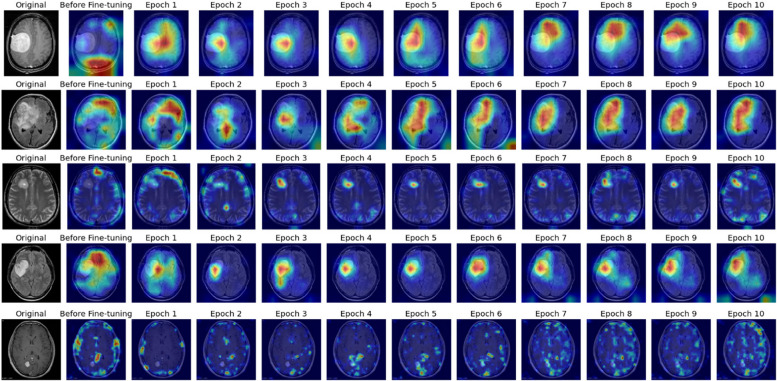
Epoch wise Gradcam

The Grad-CAM visualizations offer compelling insights into the model’s decision-making process. By spotlighting areas of focus during predictions, these visualizations validate that the model is not only learning but also focusing on the correct features within MRI images. For instance, the concentration of heatmap activations over tumor regions aligns with clinical expectations, providing a reassuring confirmation that the model’s detections are based on relevant pathological features rather than spurious correlations. To understand this more Fig. 13 enhances the visual interpretation.

In clinical practice, such visual explanations hold immense potential to enhance collaboration between AI systems and medical professionals. They facilitate a more informed and nuanced understanding of AI-assisted diagnoses, empowering clinicians to trust and effectively integrate AI tools into their diagnostic process. This ensures that the technology acts as a reliable aid rather than an opaque and uninterpretable black box. Table 7 encapsulates the loss and accuracy performance metrics of different Convolutional Neural Network (CNN) architectures when applied to the task of brain tumor detection in MRI images. These architectures are benchmarked to provide a comprehensive overview of their effectiveness, allowing for informed decisions on the optimal model for deployment in clinical settings.

**Table 7:** Comparative Performance Metrics of Various Convolutional Neural Network Architectures

CNN	Loss	0.5071
	Accuracy	80.16%
EfficientNetB0	Loss	0.4281
	Accuracy	86.51%
Densenet201	Loss	0.3953
	Accuracy	80.16%
Inception	Loss	0.3982
	Accuracy	81.75%
Xception	Loss	0.412
	Accuracy	85.71%
Mobilenet	Loss	0.3439
	Accuracy	84.13%
Proposed Model	Loss	0.0702
	Accuracy	98.30%

The data presented in Table 7 showcases the variability in performance across different deep learning models, with the Proposed architecture displaying a notable edge in accuracy. These results suggest that the Proposed model, with its distinctive approach to convolutional operations, outperforms other widely used architectures for this specific task. It is essential to note that the model’s architecture is not the sole determinant of performance; factors such as dataset complexity, data preprocessing, and augmentation strategies also play critical roles in achieving high accuracy and low loss in brain tumor detection algorithms.

### Limitations of the dataset and implications for Generalizability

The current study has employed a dataset that, while sufficiently large to train a deep learning model with high accuracy, presents certain limitations that must be addressed to understand the full scope of the model’s applicability. Notably, the dataset’s size and diversity are constrained, which may impact the model’s ability to generalize its findings beyond the scope of the study. The dataset, comprising a finite number of MRI images, is not expansive enough to encapsulate the full heterogeneity of brain tumors. Although deep learning models, such as the one we have implemented, are capable of learning complex patterns in data, their performance is inherently tied to the quantity and quality of the training data. The size of the dataset limits the model’s exposure to the wide range of variations that occur in brain tumors, potentially hindering its predictive performance in clinical scenarios that are not represented in the training data. The dataset predominantly includes MRI images from a limited demographic and may not adequately represent the diversity seen in the global population. Brain tumors vary significantly in their appearance, not only due to biological factors but also due to demographic variations. Therefore, a dataset with greater demographic diversity would likely improve the model’s generalizability and reliability across different populations. These limitations underscore the need for caution when extrapolating the study’s findings to the general population. The high accuracy and precision demonstrated by our model may not fully predict its effectiveness in a clinical setting, where the range of tumor appearances and patient backgrounds is considerably broader. Future research should focus on acquiring and incorporating a more diverse and extensive dataset that can better represent the global incidence of brain tumors. This would enable the development of a model with enhanced generalizability, more accurately reflecting the performance one might expect in diverse clinical environments.

While the present study provides valuable insights into the capabilities of deep learning for brain tumor detection, it also highlights the need for continual improvement in dataset collection and model training methodologies. By addressing these limitations, future work can lead to more robust and widely applicable diagnostic tools, ultimately contributing to improved patient care and outcomes in the domain of medical imaging.

### Ethical considerations in the deployment of AI for clinical diagnostics

As we stand on the precipice of a new era in medical diagnostics, propelled by advancements in artificial intelligence (AI), it is imperative to address the ethical considerations that accompany the deployment of such technologies. The use of sensitive patient data to train AI models demands stringent adherence to privacy regulations such as the Health Insurance Portability and Accountability Act (HIPAA) and the General Data Protection Regulation (GDPR). We advocate for robust de-identification processes to ensure that patient data remains confidential and secure, thereby upholding the privacy of individuals. Protecting the data from unauthorized access and breaches is crucial. The deployment of AI in clinical settings must be accompanied by state-of-the-art cybersecurity measures to safeguard against potential data leaks, ensuring the security and integrity of patient information. While AI has the potential to significantly improve diagnostic accuracy, there remains the risk of misdiagnosis. It is essential to establish clear protocols for human oversight, where AI acts as a decision support tool rather than a definitive diagnostician. This ensures that the ultimate responsibility for diagnosis remains with trained medical professionals, mitigating the risk of misdiagnosis due to AI errors.: Patients must be informed about the role of AI in their diagnostic process, and consent should be obtained with full transparency about the use of AI tools. This promotes trust and allows patients to make informed decisions about their healthcare. AI models can inadvertently perpetuate biases present in the training data, leading to unequal healthcare outcomes. It is critical to use diverse datasets for training and validate models across different demographics to ensure the equitable application of AI in clinical diagnostics. Post-deployment, AI systems must be continuously monitored and validated to ensure they perform as expected over time. This is especially important as AI models may degrade or become less accurate as patient populations and disease presentations evolve. The ethical deployment of AI in healthcare is a shared responsibility that requires collaboration between technologists, healthcare providers, ethicists, and policymakers. By proactively addressing these ethical concerns, we can steer the course of AI towards augmenting healthcare delivery while maintaining the highest standards of patient care and safety.

### Future research directions and clinical integration

Proposed research marks a significant step forward in the application of AI for brain tumor detection. However, the path from research to clinical implementation is multifaceted, necessitating further investigation. A primary direction for future research is the exploration of how AI tools can be seamlessly integrated into existing clinical workflows. This involves the development of user-friendly interfaces that allow radiologists to easily interact with AI predictions, the establishment of protocols for when and how AI recommendations are to be considered, and the assessment of the impact of AI tools on diagnostic accuracy and time efficiency in live clinical environments. It is imperative to conduct longitudinal studies and clinical trials to evaluate the efficacy and safety of AI-assisted diagnostics over extended periods. This will not only validate the long-term reliability of AI tools but also identify any unforeseen issues that may arise in a real-world setting. In concert with technological advancements, there is a need for developing clear regulatory and ethical guidelines that govern the use of AI in medical diagnostics. Future research should focus on contributing to policy discussions and the creation of comprehensive guidelines that ensure patient safety, data privacy, and equitable care. To advance the deployment of AI in clinical settings, interdisciplinary collaboration is essential. Future research should aim to foster partnerships between AI researchers, clinicians, ethicists, and policy-makers to ensure that the development of AI tools aligns with clinical needs and ethical standards. Preparing the next generation of healthcare providers to work alongside AI is critical. Future research should also focus on educational programs and training modules that equip medical professionals with the necessary skills to effectively utilize AI in their practice. Finally, research should continue to advance the technology itself, improving the accuracy, interpretability, and generalizability of AI models. This includes the exploration of novel AI architectures, the development of more advanced interpretability techniques, and the expansion of datasets to include a wider array of pathologies and patient demographics. The future of AI in medical diagnostics is a promising yet complex journey. By setting clear research trajectories, we can ensure that our advancements in AI not only push the boundaries of technology but are also thoughtfully and effectively translated into improved clinical care. This entails not only a deep understanding of the technology but also a conscientious effort to align with clinical goals, ethical considerations, and regulatory requirements, ultimately leading to the delivery of better patient outcomes.

The application of a deep learning model like ResNet50, augmented with data augmentation techniques and complemented by Grad-CAM for interpretability, presents a powerful tool for brain tumor detection from MRI images. The model’s high performance, coupled with the transparency provided by Grad-CAM, not only advances the field of medical imaging analysis but also paves the way for more widespread acceptance and use of AI in clinical settings.

## Conclusion

This study presents the promising application of a deep learning model, particularly ResNet50 augmented with Grad-CAM, for brain tumor detection in MRI images. Achieving a testing accuracy of 98.52% alongside high precision and recall metrics underscores the model’s efficacy in identifying brain tumors accurately. Leveraging data augmentation techniques significantly bolstered the model’s robustness and generalization capabilities across diverse imaging scenarios. Moreover, the integration of Grad-CAM provided valuable insights into the model’s decision-making process by highlighting relevant areas within the images that influenced its predictions, crucial for building trust and interpretability in medical AI applications. Despite these promising results, several limitations and areas for improvement are recognized. Firstly, the study acknowledges the relatively limited dataset size and diversity, emphasizing the need for larger and more varied datasets encompassing a broader spectrum of tumor presentations. Exploring alternative architectures like EfficientNet or DenseNet could offer insights into optimizing model complexity and computational efficiency. Additionally, future research directions include clinical validation to ensure alignment with expert assessments and integration into real-world clinical workflows to evaluate diagnostic impact and patient outcomes. Refining explainability methods such as Grad-CAM and integrating multimodal data sources could further enhance the model’s diagnostic capabilities and foster trust among medical professionals. Overall, while this study marks a significant advancement in AI-driven brain tumor detection in MRI images, ongoing research efforts aim to enhance accuracy, interpretability, and clinical applicability, paving the way for improved patient care in medical imaging analysis.

## Data Availability

The data that support the findings of this study are openly available at https://www.kaggle.com/datasets/navoneel/brain-mri-images-for-brain-tumor-detection.
